# Relationship between proximal aortic stiffness assessed with CMR and left ventricular diastolic function in a bicentric asymptomatic population with preserved left ventricular ejection fraction

**DOI:** 10.1186/1532-429X-13-S1-P378

**Published:** 2011-02-02

**Authors:** Alban Redheuil, Elie Mousseaux, Wen-Chung Yu, Nadjia Kachenoura, Ludivine Perdrix, Elzbieta Chamera, David Bluemke, Joao Lima

**Affiliations:** 1 European Hospital Georges Pompidou and INSERMU678, Paris, France; 2 Taipei Veterans General Hospital, Division of Cardiology, Taipei, Taiwan; 3 Johns Hopkins University, Baltimore, MD, USA; 4 Radiology and Imaging Sciences, National Institutes of Health, Bethesda, MD, USA

## Objective

To evaluate the direct relation between proximal aortic function and left ventricular diastolic function in individuals with normal LVEF free of overt cardiovascular disease.

## Background

Left ventricular (LV) diastolic dysfunction is considered to be an important determinant of heart failure with preserved LV ejection fraction (LVEF). Hypertension, diabetes and aging leading to increased aortic stiffness have been related to impaired diastolic function. Novel CMR measures of proximal aortic function have been recently shown to be better markers of vascular aging than conventional measures of arterial stiffness but their relationship to LV diastolic function has not been reported.

## Methods

We studied 144 subjects (70 men, 74 women, age 44±16 [13-79] years) from 2 academic centers (Baltimore, USA; Paris, France). Ascending aortic strain was determined by CMR using an automated segmentation of SSFP cine acquisitions. Aortic arch pulse wave velocity (PWV) was measured by CMR from phase-contrast sequence for flow-wave analysis and multiple T1 spin echo images for aortic length measurement. Conventional LV diastolic function indices were measured by echo-Doppler (E/A, e’, E/e’); carotid pressures and carotid-femoral PWV (cfPWV) were measured using applanation tonometry. Central pressures were used to calculate aortic distensibility and carotid augmentation index (AIx).

## Results

**Figure 1:**
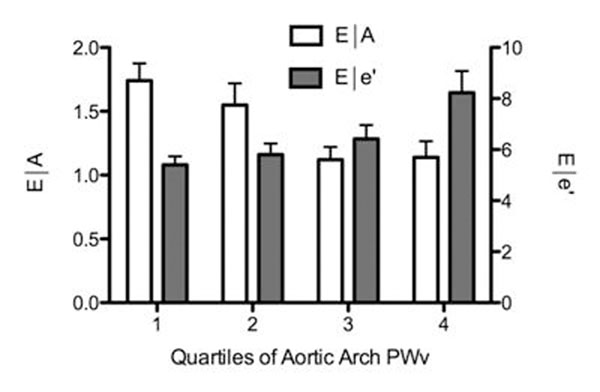
Relationship between E/A and E/e’ ratios and aortic arch PWV

As shown on Figure 1, increased aortic arch stiffness was associated with decreased E/A ratio, decreased mitral annular e’ velocity and increased E/e’ ratio. In multivariate linear regression analysis we found an independent association between impaired ascending aortic distensibility and decreased E/A ratio (p=0.01, R2=0.42) and e’ velocities (p=0.006, R2=0.62). In addition, we found an independent association between increased aortic arch PWV and increased E/e’ ratio (p<0.001, R2=0.46). These relationships were independent of age, gender, body mass index and mean central blood pressure. Moreover, these associations remained significant after further adjustment for LV mass. Similar trends were observed using central pulse pressure, AIx and cfPWV as markers of arterial stiffness but reached lower degrees of statistical significance compared to proximal markers of aortic function.

## Conclusions

Proximal aortic function measured by CMR is strongly associated with subclinical impairment in diastolic left ventricular function in subjects with preserved LVEF independent of the effects of age, gender, BMI, mean central pressure and LV mass. These novel markers of proximal aortic stiffness estimated by CMR have a stronger correlation with LV diastolic function than conventional markers of arterial stiffness.

